# Isolation and characterization of the *TaSnRK2*.*10* gene and its association with agronomic traits in wheat (*Triticum aestivum* L.)

**DOI:** 10.1371/journal.pone.0174425

**Published:** 2017-03-29

**Authors:** Zhao-Gui Zhang, Guang-de Lv, Bing Li, Jia-Jia Wang, Yan Zhao, Fan-Mei Kong, Ying Guo, Si-Shen Li

**Affiliations:** 1 State Key Laboratory of Crop Biology/Shandong Key Laboratory of Crop Biology, Shandong Agricultural University, Tai’an, China; 2 Tai’an Academy of Agricultural Science, Tai’an, China; Instituto Agricultura Sostenible, SPAIN

## Abstract

Sucrose non-fermenting 1-related protein kinases (SnRKs) comprise a major family of signaling genes in plants and are associated with metabolic regulation, nutrient utilization and stress responses. This gene family has been proposed to be involved in sucrose signaling. In the present study, we cloned three copies of the *TaSnRK2*.*10* gene from bread wheat on chromosomes 4A, 4B and 4D. The coding sequence (CDS) is 1086 bp in length and encodes a protein of 361 amino acids that exhibits functional domains shared with SnRK2s. Based on the haplotypes of *TaSnRK2*.*10-4A* (Hap-4A-H and Hap-4A-L), a cleaved amplified polymorphic sequence (CAPS) marker designated *TaSnRK2*.*10-4A-CAPS* was developed and mapped between the markers *D-1092101* and *D-100014232* using a set of recombinant inbred lines (RILs). The *TaSnRK2*.*10-4B* alleles (Hap-4B-G and Hap-4B-A) were transformed into allele-specific PCR (AS-PCR) markers *TaSnRK2*.*10-4B-AS1* and *TaSnRK2*.*10-4B-AS2*, which were located between the markers *D-1281577* and *S-1862758*. No diversity was found for *TaSnRK2*.*10-4D*. An association analysis using a natural population consisting of 128 winter wheat varieties in multiple environments showed that the thousand grain weight (TGW) and spike length (SL) of Hap-4A-H were significantly higher than those of Hap-4A-L, but pant height (PH) was significantly lower.

## Introduction

Wheat (*Triticum aestivum* L.) is one of the most important food crops worldwide, and obtaining higher yields is one of the primary objectives for wheat improvement. A large number of quantitative trait loci (QTLs) have been reported to control grain yield and yield components [1–7]. Recently, several yield-related genes have been cloned and transformed into functional markers (FMs), such as *TaGW2* [8], *TaSus2* [9], *TaCwi-A1* [10], and *TaGS1a* [11] etc. The FMs derived from polymorphic sites in genes are important for marker-assisted selection (MAS) in breeding programs [12].

Sucrose non-fermenting 1-related protein kinases (SnRKs) form a major family of signaling proteins in plants and include three gene subfamilies, *SnRK1*, *SnRK2* and *SnRK3* [13]. *SnRK1* genes play an important role in the regulation of carbon metabolism and energy status [14–15], and *SnRK3* genes encode CBL-interacting protein kinases, which specifically interact with calcineurin B-like proteins (CBLs) [16]. The *SnRK2* genes represent a group of plant-specific protein kinases that have been shown to be involved in abiotic stress signal transduction, nutrient utilization and growth in plants [17]. Ten members of the *SnRK2* gene family have been identified [15].

In wheat, *SnRK2s* are involved in the response to abiotic stress and have potential functions in carbohydrate and energy metabolism [18]. *PKABA1* was the first gene of the *SnRK2* family cloned in wheat and is induced by abscisic acid (ABA) and hyperosmotic stress [19–20]. Overexpression of *TaSnRK2*.*4* in *Arabidopsis* resulted in increased tolerance to osmotic stress, delayed seedling establishment, longer primary roots, and higher yields under both normal and stress conditions [21]. Functional analysis showed that *TaSnRK2*.*7* is involved in carbohydrate metabolism as well as decreasing osmotic potential, enhancing photosystem II activity, and promoting root growth [18]. *TaSnRK2*.*8* may participate in ABA-dependent signal transduction pathways, and overexpression of this gene results in enhanced tolerance to abiotic stress. Additionally, *TaSnRK2*.*8* transgenic plants show significantly lower levels of total soluble sugar under normal growing conditions, which suggests that this gene might be involved in carbohydrate metabolism [22]. Two other members of the *SnRK2s* found in wheat, *TaSnRK2*.*3* and *W55a*, also play important roles in the response to abiotic stress and plant growth [7, 23].

The objectives of this study were to isolate the full-length cDNA and gDNA sequences of *TaSnRK2*.*10* in wheat, to develop and map the functional markers, and to conduct an association analysis between *TaSnRK2*.*10* haplotypes and agronomic traits using a natural population of 128 varieties.

## Materials and methods

### Plant materials

Plant materials in this study came from four groups: 1) ten winter wheat varieties, including Chinese Spring, Jinan 17, Jining 17, Lumai 21, Lumai 23, Shannong 0431, Shannong 8355, Weimai 8, Xiaoyan 81, and Yannong 15, were used for the isolation of *TaSnRK2*.*10* DNA sequences and for haplotype analysis. This material was highly polymorphic and was selected from each subgroup of the 128 natural populations of varieties (NPVs) analysed using 91 SSR and 47 functional markers; 2) a set of Chinese Spring nullisomic-tetrasomic lines (CS-N/Ts) was used for determining the special chromosomes of *TaSnRK2*.*10*; 3) a set of 179 recombinant inbred lines (RILs) derived from ‘Shannong 0431 × Lumai 21’ was employed for linkage analysis. Shannong 0431 is a germplasm developed by our group with a large grain size and multi-disease resistance (wheat stripe rust, leaf rust, powdery mildew and sharp eyespot), Lumai 21 is a cultivar released by the Yantai Academy of Agricultural Science of China in 1996 and has a high yield and high drought resistance; and 4) a natural population of varieties (NPVs) was employed to validate the functional markers and analyze the relationships between *TaSnRK2*.*10* haplotypes and agronomic traits. The population consisted of 128 winter wheat varieties released in the Huang-huai Winter Wheat Region and the Northern Winter Wheat Region of China.

### DNA and RNA extraction and first-strand reverse transcription of cDNA

After sterilization for 5 min in a 10% solution of H_2_O_2_ and washing three times with sterilized water, wheat seeds were germinated and cultured in a growth chamber (20±1°C with 12 h light, 12 h dark cycle). Ten days later, wheat leaves were sampled for the isolation of gDNA and total RNA. The gDNA was extracted from lyophilized mixed leaves using the CTAB method [24]. The RNA was extracted using TRIzol reagent (Invitrogen Co., Ltd., Shanghai, China), and the first-strand synthesis was performed using M-MLV transcriptase (Invitrogen Co., Ltd., Shanghai, China) according to the manufacturer’s instructions.

### Cloning, sequence analysis and development of genome-specific primers

To obtain the sequence of *TaSnRK2*.*10*, the cDNA sequence of *SAPK10* from rice (GenBank ID: AB125311) was used as a query sequence to screen the GenBank wheat EST database. All candidate ESTs showing high similarity to *SAPK10* cDNA were obtained through BLASTN searches (http://www.ncbi.nlm.nih.gov) and assembled into a putative *TaSnRK2*.*10* cDNA sequence using the CAP3 Sequence Assembly Program (http://doua.prabi.fr/software/cap3). The functional region and activity sites were identified with PROSITE (http://prosite.expasy.org/). The primer pairs for *TaSnRK2*.*10-1F/R* and *TaSnRK2*.*10-2F/R* (Table 1) were designed based on the putative sequence using Primer Premier Version 5.0 software (http://www.premierbiosoft.com/) and were used for isolating the cDNA and gDNA sequences of *TaSnRK2*.*10*. The genome-specific primer pairs for *TaSnRK2*.*10-3-4AF/R*, *TaSnRK2*.*10-3-4BF/R* and *TaSnRK2*.*10-3-4DF/R* (Table 1) were designed based on DNA sequence variations among the genomic sequences to identify homoeologs as well as specific alleles at individual loci.

**Table 1:** Primers used in this study.

Primer set	Primer sequence (5'-3')	Amplified target	Size (bp)
TaSnRK2.10–1	Forward: GCTTGCTCGGTTGCTTTGC	*TaSnRK2*.*10*cDNA	1339, 1342,and 1284
	Reverse: CATCCAAAAGGCCAAACCGT		
TaSnRK2.10–2	Forward: GTCAAGTACATCGAGCGAGGG	*TaSnRK2*.*10*gDNA	2127 or 2130, 2052 and 2076
	Reverse: GTCGGCGTCTGAATCAAGGT		
TaSnRK2.10-3-4A	Forward: CTTCATTCGCAACCAAAATCTACG	A genome-specific	1109 or1106
	Reverse: GAACTGGTTGATCCGAGAACCG		
TaSnRK2.10-3-4B	Forward: GCTTGCTTCACTGTCGCAG	B genome-specific	688
	Reverse: GCAGAGTCTAGCAGTACCGTT		
TaSnRK2.10-3-4D	Forward: CCATGACGTTCTCCGTTCCC	D genome-specific	1296
	Reverse: GCACACTCAATATCCTCTGGC		
TaSnRK2.10-4A-CAPS	Forward: CTTCATTCGCAACCAAAATCTACG		1109 or 1106
	Reverse: GAACTGGTTGATCCGAGAACCG		
TaSnRK2.10-4B-AS1	Forward: GCTTGCTTCACTGTCGCAGG		688
	Reverse: GCAGAGTCTAGCAGTACCGTT		
TaSnRK2.10-4B-AS2	Forward: GCTTGCTTCACTGTCGCAGA		688
	Reverse: GCAGAGTCTAGCAGTACCGTT		

PCR assays were performed using LA Taq polymerase (TaKaRa Biotechnology Co., Ltd., Dalian, China) in a 20 μL reaction mixture containing 80 ng of gDNA or cDNA, 5 pM of *TaSnRK2*.*10-1F/R* or *TaSnRK2*.*10-2F/R*, 200 μM of each dNTP, 1 unit of LA Taq and 2 μL of 10× PCR buffer. A touchdown PCR procedure was employed as follows: initial denaturation at 95°C for 5 min, followed by 10 amplification cycles of 35 s at 95°C, 35 s at 63°C with a decrease of 0.5°C per cycle and 2 min at 72°C, followed by 30 amplification cycles of 30 s at 95°C, 45 s at 59°C and 2 min at 72°C, and a final extension step at 72°C for 10 min. The PCR products were separated on 1.0% agrose gels, and the target bands were recovered with the TIANgel Midi Purification kit (TianGen Biotech Co., Ltd., Beijing, China) and cloned into the pEASY-T1 simple vector (TransGen Biotech Co., Ltd., Beijing, China) before being transformed into competent *E*. *coli DH5α* cells via the heat shock method. Positive clones were selected for sequencing by Sangon Biotechnology Co. Ltd. (Shanghai, China). Using the software DNAMAN (http://www.lynnon.com/), the positions of exons and introns in the *TaSnRK2*.*10* gene were determined by aligning the amplified gDNA and the corresponding cDNA sequences. The sequence alignment and similarity to other species were determined using the NCBI database. A phylogenetic tree was constructed based on the full-length amino acid sequences of SnRK2s using the protein sequences aligned by MAFFT7 [25]. The maximum-likelihood phylogenetic tree was reconstructed using MEGA5 [26], and the phylogenetic support for each split was evaluated with 500 bootstrap replicates.

### Development and location of functional markers

We analysed the sequence of the coding region for gene *TaSnRK2*.*10* in the ten winter wheat varieties and found two haplotypes for *TaSnRK2*.*10-4A* and *TaSnRK2*.*10-4B*, respectively. Using the Primer Premier 5.0 software, the polymorphic site for distinguishing the haplotypes of the *TaSnRK2*.*10* gene were transformed into a cleaved amplified polymorphism sequence (CAPS) and allele-specific PCR (AS-PCR) markers [27] for *TaSnRK2*.*10-4A-CAPS* and *TaSnRK2*.*10-4B-AS1/AS2* (Table 1), respectively. The primer pairs were used to amplify the genome-specific *TaSnRK2*.*10* allele of CS-N/Ts, RILs and NPVs through PCR. PCR was performed using the following program: 95°C for 5 min, followed by 30 cycles of 95°C for 30 s, 60°C for 30 s, and 72°C for 1 min, and then a final extension of 72°C for 10 min. The PCR products for the CAPS marker were digested with *Sal*I (TaKaRa Biotechnology Co., Ltd., Dalian, China) according to the manufacturer’s directions. All segments were separated on 1.0% agarose gels with EB. For location of the functional markers a genetic map of RILs was used which was constructed using SSR markers and DArT array of Wheat PstI (TaqI) 2.6 and Wheat GBS 1.0 (Triticarte Pty. Ltd, Canberra, Australia) (Unpublished data).

### Measurements of agronomic traits and association analysis

The phenotypes of the natural population of 128 wheat varieties were evaluated in field trials in three environments: Tai’an 2011 (TA11), Tai’an 2012 (TA12) and Yan’tai 2012 (YT12), in Shandong Province, China. Tai’an is part of the Huang-Huai Winter Wheat Region and Yantai is part of the Northern Winter Wheat Region of China. Each plot consisted of 3 rows that were 1.5 m long and spaced 25 cm apart; 70 seeds were planted in each row with two replicates. Plant height (PH), grain number per spike (GNS), spike length per plant (SL), sterile spikelet number per spike (SSS), fertile spikelet number per spike (FSS) and total spikelet number per spike (TSS) were determined from 10 random spikes for each line in each replicate at the grain-filling stage. The fertile spikelet number per spike (FSS) was calculated as TSS minus SSS. A 50 cm uniformed row was chosen to measure the spike number per plant (SN). The thousand grain weight (TGW) was evaluated by weighing three samples of 200 grains from each plot after harvest.

The unified mixed linear model (MLM) based on the Q + K model was used for functional markers and agronomic traits analysis in TASSEL v.2.0.1 [28–29]. The population structure matrix (Q) was obtained using STRUCTURE 2.3.1 software [30]. The relative kinship matrix (K) was obtained using TASSEL software [31]. Corrections for multiple testing were performed using the positive FDR (FDR ≤ 0.1) in QVALUE [32]. The 91 SSR and 47 functional markers were used to calculate Q and K for NPVs. A connection between functional markers and agronomic traits was determined when *P* ≤ 0.05.

## Results

### Cloning, chromosome assignment and characterization of *TaSnRK2*.*10*

Four wheat ESTs (CJ827375, CD882003, CD918384 and BJ294918) similar to the cDNA sequence of *SAPK10* were selected and assembled into a putative *TaSnRK2*.*10* cDNA sequence. Three cDNA clones were amplified with the *TaSnRK2*.*10-1F/R* primer pair, and the corresponding gDNA sequences were amplified with the *TaSnRK2*.*10-2F/R* primer pair (S1–S3 Figs). Based on the three genome-specific primer pairs, *TaSnRK2*.*10* genes were found to be located on chromosomes 4A, 4B and 4D using the CS-N/Ts (S4 Fig). The sequence of *TaSnRK2*.*10* is shared high sequence similarity with the currently most updated gene model in Chinese Spring (http://plants.ensembl.org/Triticum_aestivum/Gene), including the uniformity of 99% for *TaSnRK2*.*10-4A* with TRIAE_CS42_4AL_TGACv1_291776_AA0996560, 100% for *TaSnRK2*.*10-4B* with TRIAE_CS42_4BS_TGACv1_328137_AA1083460, and 99% for *TaSnRK2*.*10-4D* with TRIAE_CS42_4DS_TGACv1_361281_AA1164930.

**Fig 1:**
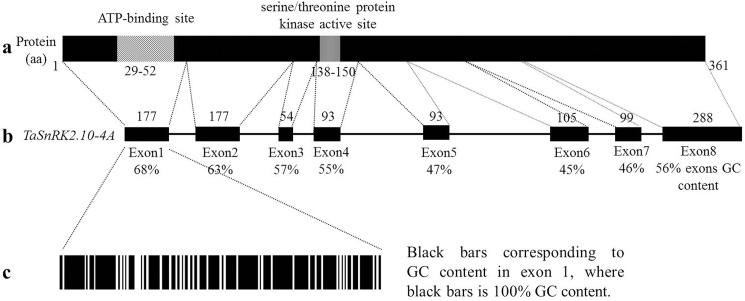
Schematic diagram of the *TaSnRK2*.*10* gene. (a) Known functional domains of *TaSnRK2*.*10* protein are highlighted; ATP-binding site in diagonal stripes, serine/threonine protein kinase active site in vertical stripes. Amino acid positions of functional domains are indicated below the protein structure. (b) Exon-intron structure of *TaSnRK2*.*10-4A* gene with its eight coding exons in black boxes; the number of base pair sequence of ORF is listed above each exon; The percentage of GC content for exon 1–8 is indicated below each exon. (c) The GC content in exon 1.

The cDNA sequences of *TaSnRK2*.*10-4A*, *TaSnRK2*.*10-4B* and *TaSnRK2*.*10-4D* amplified with the *TaSnRK2*.*10-1F/R* primer pair are 1339, 1342 and 1284 bp in length, respectively. Each cDNA sequence of *TaSnRK2*.*10* on 4A, 4B and 4D contained an open reading frame (ORF) of 1086 bp through ORF finder (https://www.ncbi.nlm.nih.gov/orffinder/) which was predicted to encode a protein of 361 amino acid residues (AARs) (Fig 1) with a molecular mass of ~40.6 kDa and a pI of ~4.80. PROSITE analysis indicated that the amino acid sequence contains two conserved domains. The first conserved domain is an N-terminal catalytic domain (23–279 downstream of the Met) containing an ATP-binding site (29–52 downstream of the Met) and a serine/threonine protein kinase active site (138–150 downstream of the Met) (Fig 1). The second domain is a relatively short C-terminal domain with abundant Asp (D) residues. The amino acid sequence of *TaSnRK2*.*10* shared high sequence similarity with counterpart monocot SnRK2s, including 95.6% with SAPK10 from rice and 94.2% with ZmSnRK2.10 from maize, and lower sequence similarity with dicotyledonous plants, including 62.5% with AtSnRK2.10 from *Arabidopsis*. The phylogenetic tree of the TaSnRK2.10 and SnRK2 family members from *Arabidopsis*, rice and maize showed that TaSnRK2.10 clustered in the same clade as OsSAPK10 and ZmSnRK2.10 (S5 Fig).

**Fig 2:**
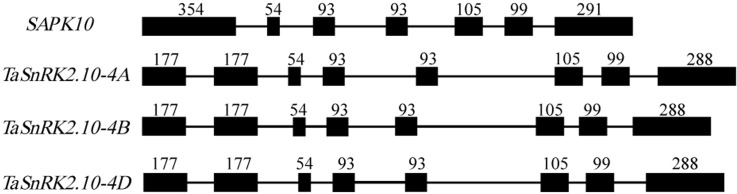
**The structures of the *TaSnRK2*.*10* gene from 4A, 4B and 4D homoeologs in wheat.** The black boxes denote exons, and the lines between exons represent introns. The numbers upon exons indicate their size (bp).

The complete gDNA sequences (from the ATG start codon to the TGA stop codon) of *TaSnRK2*.*10-4A*, *TaSnRK2*.*10-4B* and *TaSnRK2*.*10-4D* are 2322, 2244 and 2268 bp in length, respectively (S1–S3 Figs), with eight exons and seven introns (Fig 2). The exon-intron structure of *TaSnRK2*.*10* is very similar to *SnRK2*.*10* in maize and *Arabidopsis*, while the *SAPK10* gene of rice comprises seven exons and six introns. Compared with rice, the first exon of *OsSAPK10* could be divided into the first and second exon in *TaSnRK2*.*10* (Fig 2), but the sizes of the other exons are similar between wheat and rice, showing high sequence identity even though the introns exhibit low sequence similarity.

### Development and mapping of the functional markers

For the *TaSnRK2*.*10* gDNA sequences among the ten wheat varieties, three SNPs (single nucleotide polymorphisms) and one indel (insertion or deletion of DNA bases) were found for *TaSnRK2*.*10-4A* with two haplotypes (named Hap-4A-H and Hap-4A-L). The SNPs were located at 1273, 1471, and 1907 bp downstream of the ATG with corresponding C-T, A-T, and G-C substitutions, respectively. The indel was 3 bp (TGA, 1982–1984 bp downstream of the ATG) (S1 Fig). For *TaSnRK2*.*10-4B*, one SNP was located at 856 bp downstream of the ATG with A-G substitution, and formed two haplotypes (named Hap-4B-A and Hap-4B-G) (S2 Fig). No variation in *TaSnRK2*.*10-4D* was observed.

**Fig 3:**
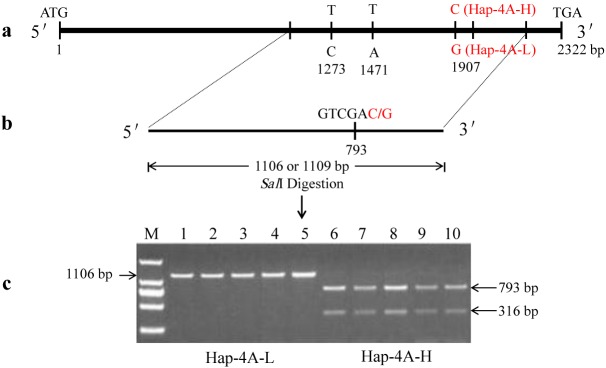
Functional marker development based on a SNP found in the seventh exon of *TaSnRK2*.*10-4A*. (a) The positions of the Hap-4A-H and Hap-4A-L SNPs in *TaSnRK2*.*10-4A*. (b) SNP1907 (C-G) in the PCR product permitted the generation of different *Sal*I restriction fragments with lengths of 793 and 316 bp in the varieties harboring Hap-4A-H, while no digestion product was obtained in Hap-4A-L. (c) Validation of CAPS in varieties with Hap-4A-L (1–5) and Hap-4A-H (6–10) on 1.0% agarose gel. M, marker; 1–5 are the varieties Lumai 21, Jinan 17, Yannong 15, Chinese Spring, and Xiaoyan 81, respectively; and 6–10 are the varieties Shannong 0431, Jining 17, Shannong 8355, Lumai 23 and Weimai 8, respectively.

**Fig 4:**
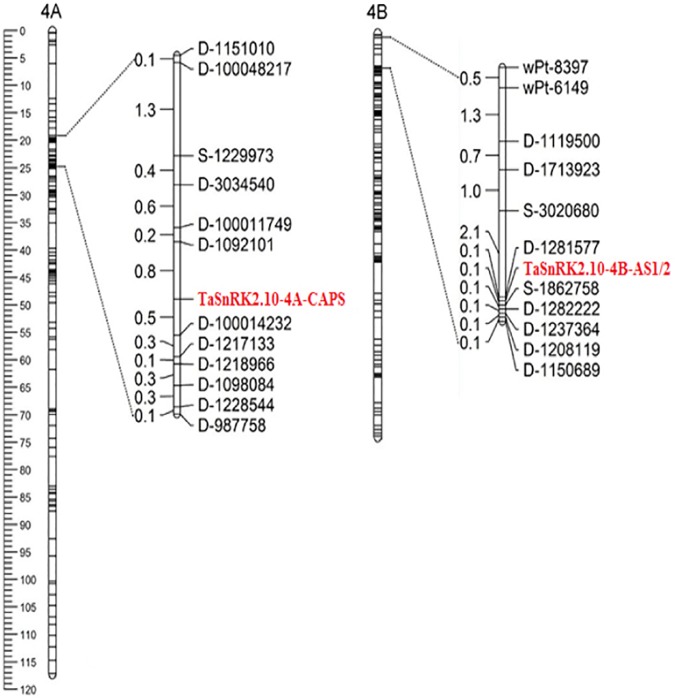
Mapping of *TaSnRK2*.*10-4A* and *TaSnRK2*.*10-4B* based on a RIL population of Shannong 0431×Lumai 21.

The sequence variations of *TaSnRK2*.*10-4A* produced a restriction enzyme *Sal*I recognition site (GTCGAC) in Hap-4A-H at SNP-1907-C, but not in Hap-4A-L at SNP-1907-G (GTCGAG) (Fig 3A and 3B). Based on this SNP, a CAPS marker *TaSnRK2*.*10-4A-CAPS* (Table 1) was developed to distinguish the *TaSnRK2*.*10-4A* allele. The PCR product for Hap-4A-H was digested by *Sal*I into two segments of 793 and 316 bp (Fig 3C). The RILs were genotyped using *TaSnRK2*.*10-4A-CAPS*, and the mapping result using RILs showed that it had highly linked two Diversity Arrays Technology (DArT) markers, *D-1092101* and *D-100014232*, with 0.80 and 0.50 cM, respectively (Fig 4).

**Fig 5:**
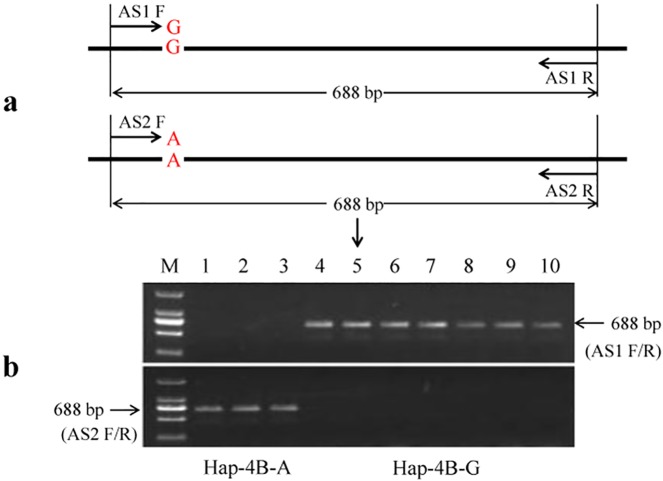
Functional marker development based a SNP found in the fourth intron of *TaSnRK2*.*10-4B*. (a) Schematic diagram showing the AS-PCR approach used tovalidate the SNP. (b) PCR fragments amplified with the markers *TaSnRK2*.*10-4B-AS1* and *TaSnRK2*.*10-4B-AS2* in ten Chinese wheat varieties. *TaSnRK2*.*10-4B-AS1* yielded a 688 bp PCR fragment in varieties with the Hap-4B-G allele, and *TaSnRK2*.*10-4B-AS2* yielded a 688 bp fragment in those with the Hap-4B-A allele. M, marker, 1–3 are the varieties Lumai 21, Jinan 17, Yannong 15, respectively; and 4–10 are the varieties Chinese Spring, and Xiaoyan 81, Shannong 0431, Jining 17, Shannong 8355, Lumai 23 and Weimai 8, respectively.

Two complementary dominant AS-PCR markers were developed for *TaSnRK2*.*10-4B* (Table 1). The primer pair *TaSnRK2*.*10-4B-AS1F/R* yielded a 688 bp PCR fragment for Hap-4B-G, but no PCR fragment for the Hap-4B-A allele. The primer pair *TaSnRK2*.*10-4B-AS2F/R* yielded a 688 bp fragment for Hap-4B-A, but no PCR fragment for the Hap-4B-G allele (Fig 5). The mapping result using RILs showed that *TaSnRK2*.*10-4B-AS1/2* was highly linked to the DArT marker *D-1281577* and the SNP marker *S-1862758* with 0.10 and 0.10 cM, respectively (Fig 4).

### Association between haplotypes and agricultural traits

Data on agricultural traits for the natural population of 128 wheat varieties were used in an association analysis. Using the *TaSnRK2*.*10-4A-CAPS* marker, 63 varieties harboring the Hap-4A-H haplotype and 65 varieties with the Hap-4A-L haplotype were identified. The TGW of Hap-4A-H was significantly higher than Hap-4A-L in all three environments as well as the average value (AV) at the *p*≤0.01 level. The PH of Hap-4A-L was significantly higher than Hap-4A-H in TA11, YT12 and the AV (*p*≤0.05), but SL was lower for TA11, TA12 and the AV (*p*≤0.05). The TSS of Hap-4A-H was significantly higher for TA12 and the AV as well as SSS in the AV (*p*≤0.05). These results indicated that the environments are important in explaining the overall phenotypic variations. Using *TaSnRK2*.*10-4B-AS1* and *TaSnRK2*.*10-4B-AS2* markers, 93 varieties with Hap-4B-G haplotypes and 35 varieties with Hap-4B-A haplotypes were identified. However, there were no significant differences between the haplotypes except for TGW in TA11 (*p* = 0.025) (Table 2).

**Table 2:** Association analysis between the haplotypes and agronomic traits.

Trait	ENV	Hap-4A-H	Hap-4A-L	*p* value	Hap-4B-G	Hap-4B-A	*p* value
TGW	TA11	53.161 ± 5.505	51.189 ± 5.644	0.003**	52.509±5.835	51.325±5.098	0.025*
(g)	TA12	46.349 ± 5.967	43.554 ± 4.881	0.007**	45.342±6.104	43.816±3.875	0.155
	YT12	47.101 ± 5.140	45.373 ± 3.998	0.010**	46.397±4.880	45.594±3.978	0.190
	AV	48.870 ± 4.989	46.705 ± 3.934	0.002**	48.083±4.892	46.912±3.684	0.051
KNS	TA11	44.661 ± 7.009	42.894 ± 5.179	0.242	43.925±6.271	43.243±6.093	0.663
	TA12	46.347 ± 7.795	44.148 ± 6.025	0.427	45.513±7.423	44.754±5.938	0.739
	YT12	59.721 ± 9.037	56.657 ± 7.805	0.324	58.976±8.242	56.394±8.902	0.469
	AV	50.243 ± 6.740	47.900 ± 4.381	0.193	49.438±5.743	48.131±5.835	0.911
PH	TA11	64.908 ± 7.136	67.194 ± 11.303	0.018*	66.615±10.529	64.721±6.003	0.706
(cm)	TA12	79.985 ± 7.853	80.417 ± 11.612	0.220	81.292±10.810	77.345±6.288	0.722
	YT12	70.115 ± 8.763	72.936 ± 12.463	0.031*	72.229±12.212	69.766±5.715	0.838
	AV	71.669 ± 7.266	73.516 ± 11.348	0.041*	73.378±10.672	70.611±5.559	0.909
SL	TA11	8.923 ± 1.068	8.510 ± 1.062	0.014*	8.756±1.708	8.595±1.111	0.344
(cm)	TA12	9.036 ± 1.164	8.565 ± 0.971	0.020*	8.845±1.153	8.631±0.898	0.638
	YT12	9.322 ± 1.149	9.071 ± 1.136	0.411	9.206±1.191	9.183±1.038	0.620
	AV	9.094 ± 1.036	8.715 ± 0.964	0.045*	8.936±1.047	8.803±0.940	0.797
TSS	TA11	19.905 ± 1.489	19.364 ± 1.270	0.081	19.839 ± 1.352	19.063 ± 1.425	0.066
	TA12	20.946 ± 1.693	20.047 ± 1.431	0.017*	20.709 ± 1.679	19.856 ± 1.301	0.164
	YT12	19.705 ± 1.331	19.383 ± 1.143	0.359	19.646 ± 1.312	19.282 ± 1.020	0.397
	AV	20.185 ± 1.352	19.598 ± 1.096	0.048*	20.065 ± 1.292	19.401 ± 1.056	0.110
FSS	TA11	2.023 ± 0.808	2.007 ± 0.858	0.769	2.218±0.817	1.713±0.816	0.062
	TA12	2.429 ± 1.204	2.305 ± 1.220	0.490	2.505±1.272	1.996±0.957	0.085
	YT12	0.457 ± 0.421	0.474 ± 0.474	0.813	0.477±0.465	0.424±0.469	0.872
	AV	1.636 ± 0.637	1.595 ± 0.673	0.785	1.704±0.646	1.378±0.632	0.072
SSS	TA11	17.882 ± 1.644	17.357 ± 1.135	0.068	17.711±1.464	17.350±1.332	0.476
	TA12	18.517 ± 1.768	17.742 ± 1.398	0.077	18.204±1.677	17.860±1.497	0.961
	YT12	19.248 ± 1.362	18.909 ± 1.165	0.342	19.169±1.364	18.859±1.105	0.355
	AV	18.549 ± 1.343	18.003 ± 0.888	0.045*	18.362±1.187	18.022±1.093	0.485

The natural population of 128 varieties was grouped according to their *TaSnRK2*.*10-4A* haplotypes (Hap-4A-H and Hap-4A-L) and *TaSnRK2*.*10-4B* haplotypes (Hap-4B-G and Hap-4B-A). The presented values are the mean ± SD from the association analysis.

* and ** designate significance differences at *p*≤0.05 and *p*≤0.01, respectively.

## Discussion

A major hindrance to PCR amplification of GC-rich templatesis the formation of secondary structures such as hairpin loops of single-stranded GC-rich sequences [33–34]. Many approaches have been developed to overcome such problems by adjusting the PCR procedure [34–39]. However, it is also difficult to get GC-rich sequence using the conventional RACE technique. In recent years, a great number of wheat ESTs have been deposited, which makes it possible to clone the full-length sequences with GC-rich sequences in combination ESTs with PCR amplification. In our present study, four EST sequences were found by performing a BLAST search with a reference sequence and were then combined to generate the tentative full-length sequence of *TaSnRK2*.*10*. The size of *TaSnRK2*.*10* is in accordance with *SnRK2s* reported previously [18, 22, 40–43].

Based on the protein size and character of the acidic amino acid-enriched C-terminus, the SnRK2 family can be divided into two groups: SnRK2a and SnRK2b [44]. SnRK2a corresponds to the more recently defined subclass I, and SnRK2b includes subclasses II and III [21, 45]. Increasing evidence indicates that SnRK2s from subclass III are involved in the regulation of plant metabolism [46]. As shown in our study, TaSnRK2.10 was clustered in the subclass III clade (S5 Fig). The structure of TaSnRK2.10 is similar to other SnRK2s, including two typical domains, an N-terminal highly conserved kinase domain and a regulatory C-terminal domain [46], and showed potential for serine/threonine and tyrosine kinase activities. The relatively short C-terminal domain of SnRK2.10 is abundant in Asp (D) and might play a role in activation of the kinase [47–49] and function in protein-protein interactions that are mainly involved in ABA responsiveness [40]. In rice and maize, *SnRK2*.*10* is activated under ABA and hyperosmotic stress. In *Arabidopsis*, *AtSnRK2*.*10* was found to be expressed in the vascular tissue at the base of developing lateral roots, revealing a role in root growth and architecture [50]. The ortholog in tobacco, *NtOSAK*, has been shown to directly interact with glyceraldehyde-3-phosphate dehydrogenase (GAPDH), linking its mode of action to metabolic processes [51]. All of the above evidence implies that *TaSnRK2*.*10* is mainly involved in ABA responsiveness and shows a potential role in carbohydrate metabolism. In this study, we found that Hap-4A of *TaSnRK2*.*10* was associated stably with the TGW, PH and SL, which may indicate new functions of *SnRK2*.*10* and may be the result of carbohydrate metabolism. The Hap-4A-H varieties of *TaSnRK2*.*10-4A* showed higher TGW and SL values than the Hap-4A-L varieties, but lower PH values, indicating the Hap-4A-H is a favorable allele for the improvement of grain yield. The marker *TaSnRK2*.*10-4A-CAPS* may be useful in wheat yield breeding programs.

Exons are the regions encoding proteins in the ORFs of genes. Many studies have indicated that missense mutations can influence the function of genes. For example, Wang et al. [52] reported that the mutant *chs1-2* with a nucleotide substitution from G to A displayed defense-associated phenotypes compared with the wild type of *CHS1*, including extensive cell death, the accumulation of hydrogen peroxide and salicylic acid, and an increased expression of PR genes. Wang et al. [53] found a codon change from TGC to TAC in the first exon of *OsCESA7*, and the mutation deleteriously affected cellulose biosynthesis and plant growth. In this study, three SNPs and one indel were found in *TaSnRK2*.*10-4A* with two haplotypes. Of these, a SNP in the seventh exon in *TaSnRK2*.*10-4A* caused a missense mutation that resulted in an amino acid change from Asp to His (S1 Fig). The adjacent region is conserved between *TaSnRK2*.*10* and the ortholog genes *SAPK9* and *SAPK10* in rice [54] (S5 Fig). There has been no report about the change of the function for the amino acid from Asp to His in *SnRK2* gene family. The missense mutation may account for the variance in agronomic traits and this should be affirmed by more evidences in the further. The other SNPs and the indel were located in introns, which may be of little function for agronomic traits. Furthermore, only one SNP was obtained for *TaSnRK2*.*10-4B* in an intron, and it had no significant association with agronomic traits except for the TGW data in TA11. The potential functions of *TaSnRK2*.*10-4B* require further investigation for verification.

The wheat yield is affected by many factors and is a polygenic trait influenced by environmental and genetic interactions at all stages of the plant’s growth [55]. Direct cloning of yield-related genes in hexaploid wheat was difficult due to its large genome size. In our study, we hypothesized that the *TaSnRK2*.*10* gene had functions that affect the TGW, PH and SL during the maturity stage. To date, some grain weight genes have been isolated, such as *Ppd-D1* [56], *CKX6-D1* [57], *GS1a* [11], *GW2* [8, 58–59], *GS-D1* [60], *Sus* [9, 61], *GASR7* [62–63], *TEF-7* [64], *CWI* [65], and *1-FEH-w3* [66–67]. Using sequence comparison and the analysis of protein domains with PROSITE (http://prosite.expasy.org/), we found that *TaSnRK2*.*10* is different from these genes and is a new gene for grain weight. More than 20 plant height genes in wheat were detected and only few were cloned. Zhang et al. [68] reported that the *Rht-B1* and *Rht-D1* in the fourth homologous group had functions that affect the TGW and kernel number per spike, but the sequences of them were different from *TaSnRK2*.*10*. Currently, there has been no report regarding the cloning of a gene that affects the SL. The validation of QTLs provides the critical first step for further mapping and gene cloning. Some QTLs for the TGW, PH and SL were located on chromosomes 4A, 4B and 4D [69–73]. The relationship between the *TaSnRK2*.*10* and these QTLs requires further study.

## Conclusion

A triplicate set of *TaSnRK2*.*10* homoeologs was cloned and assigned to chromosomes 4A, 4B and 4D. The corresponding full-length gDNA sequences of *TaSnRK2*.*10* were 2322, 2244, and 2268 bp, comprising eight exons and seven introns and presenting an ORF of 1086 bp that encodes a protein of 361 amino acids with functional domains shared with SnRK2s. One SNP in an exon and two SNPs and one indel in introns were detected in *TaSnRK2*.*10-4A* alleles, resulting in two haplotypes: Hap-4A-H and Hap-4A-L. In *TaSnRK2*.*10-4B* alleles, only one SNP in an intron was detected, also resulting in two haplotypes: Hap-4B-G and Hap-4B-A. The sequences of *SnRK2*.*10-4D* were completely conserved. A CAPS marker for *TaSnRK2*.*10-4A* and two AS-PCR markers for *TaSnRK2*.*10-4B* were developed and mapped on chromosomes. The results of an association analysis provided evidence that *TaSnRK2*.*10-4A* shows an association with TGW in all of the examined environments and with PH and SL in most environments, representing new functions of SnRK2s in wheat. Hap-4A-H was found to be a favorable allele for the improvement of grain yield.

## Supporting information

S1 Fig Genome DNA sequences alignment between two haplotypes of TaSnRK2.10-4A. The exons were indicated by the gray shade and the green shaded bases are SNPs and InDels between the two haplotypes.
(RAR)

S2 Fig Genome DNA sequences alignment between two haplotypes of TaSnRK2.10-4B. The exons were indicated by the gray shade and the green shaded bases are SNPs between the two haplotypes.
(RAR)

S3 Fig Genome DNA sequences alignment of TaSnRK2.10-4D. The exons were indicated by the gray shade.
(RAR)

S4 Fig Chromosomal locations of TaSnRK2.10 homoeologs based on genome-specific primer pairs using Chinese Spring nullisomic-tetrasomic lines. M, marker; 1, Shannong 0431; 2, Lumai21; 3, N4AT4B (nullisomic 4A-tetrasomic 4B); 4, N4BT4A; 5, N4DT4B; 6, H2O.
(RAR)

S5 Fig Phylogenetic tree of TaSnRK2.10 and SnRK2s from Arabidopsis, rice, maize and wheat. Three distinct isoform groups are presented within the boxes. The phylogenetic tree was constructed based on the full-length amino acid sequences of SnRK2s using the protein sequences were aligned by MAFFT7 [25]. The Maximum-likelihood phylogenetic tree was reconstructed using MEGA5 [26], and the phylogenetic support for each split was evaluated with 500 bootstrap replicates.
(RAR)
